# Aggressive treatment of early acute focal inflammatory activity to extend the window for BTK inhibition in multiple sclerosis

**DOI:** 10.1177/17562864251359002

**Published:** 2025-09-20

**Authors:** Joost Smolders, Ide Smets, Beatrijs Wokke

**Affiliations:** Department of Neurology, MS Center ErasMS, Erasmus MC, University Medical Center Rotterdam, Dr. Molewaterplein 40, 3015GD Rotterdam, The Netherlands; Department of Immunology, MS Center ErasMS, Erasmus MC, University Medical Center Rotterdam, Rotterdam, The Netherlands; Neuroimmunology Research Group, Netherlands Institute for Neuroscience, Amsterdam, The Netherlands; Department of Neurology, MS Center ErasMS, Erasmus MC, University Medical Center Rotterdam, Rotterdam, The Netherlands; Department of Neurology, MS Center ErasMS, Erasmus MC, University Medical Center Rotterdam, Rotterdam, The Netherlands

**Keywords:** Bruton’s tyrosine kinase, disease-modifying therapies, multiple sclerosis, progression, relapses

## Abstract

The development of novel therapy classes such as Bruton’s tyrosine kinase (BTK) inhibitors, which target disability progression independent of relapses and largely independent of new lesion formation, requires a reappraisal of strategies in the treatment of multiple sclerosis (MS). We argue that this novel class of treatment further emphasizes the need for early and aggressive treatment with classical disease-modifying compounds for the prevention of both relapses and new MRI lesion formation and their associated disability accrual. This will extend the window to recognize early progressive disability accumulation independent of acute focal inflammatory activity, and for people with MS to benefit from novel therapies such as BTK inhibition, which target damaging pathophysiological processes independent of peripherally driven focal inflammation.

## Plain language summary

Aggressive treatment of MS attacks to extend the window for novel therapies to halt progressive disability in multiple sclerosis

People affected by multiple sclerosis (MS) experience attacks which can result in persistent neurological disability. Additionally, a gradual and relentless neurological deterioration can be experienced over time without attacks, referred to as MS progression. MS attacks can be treated well with currently registered highly-effective MS therapies. However, these drugs have little impact on progression-associated disability. Currently, novel classes of MS therapies are appearing. These drugs have a limited impact MS attacks but do delay MS progression-associated disability in clinical studies. In this work, we argue that this novel development warrants an aggressive and active treatment of MS attacks. This approach allows detection of MS progression-associated disability independent of MS attacks. Hereby, this can create an optimal window for people with MS to benefit from these novel therapies.

## Introduction

The treatment of multiple sclerosis (MS) was revolutionized by the development of the currently registered classical disease-modifying therapies (DMTs).^1^ These drugs suppress the occurrence of disability associated with acute focal inflammation (relapses), effectively suppress the formation of new and gadolinium-enhancing MRI lesions, and improve long-term neurological functioning in many people with MS. Nevertheless, some people with MS continue to develop progressive neurological disability despite being treated with these compounds, a process being referred to as progressive MS.^2^ This process proves highly resistant to the currently registered DMTs, and it is associated with loss of independence and quality of life. Recently, new drugs targeting worsening not associated with relapses or radiological activity are finding their way from clinical trials to clinical practice. The Bruton’s tyrosine kinase (BTK) inhibitor tolebrutinib is likely to be the first drug in this class to become available.^3–5^ However, as the impact of tolebrutinib on relapses was not superior to first-line treatment teriflunomide in the clinical trials, and associated with a numerically higher rate of gadolinium-enhancing MRI lesions compared to teriflunomide, this compound risks a suboptimal treatment of worsening associated with these acute inflammatory events. This challenges MS treatment paradigms and requires a balancing act in terms of pharmacologically addressing both aspects of MS.

In this paper, we will review how relapse- or non-relapse-associated worsening can culminate in increased disability, and discuss how patient characteristics may guide efficacious application of therapeutic agents targeting these processes in MS. Ultimately, we will stress the importance of an early and aggressive treatment of peripherally driven acute focal inflammatory events as relapses and new T2 lesion formation, to extend the window for recognition of early disability accumulation independent of acute focal inflammatory activity and target underlying processes with novel therapeutic approaches such as BTK inhibition.

## The relevance of suppressing acute focal inflammatory activity with classical DMTs

Frequent occurrence of relapses in the early years after an MS diagnosis is a consolidated risk factor for future disability accumulation.^6–9^ A short interval between and poor recovery of relapses holds a risk of reaching disability milestones early,^7,8^ as does a multifocal attack localization and localization in the cerebellum or spinal cord.^8,10^ Since clinical relapses and new T2 lesions are both events driven by focal inflammation mediated by infiltrating leukocytes, both show a similar association with future disability. The radiological factors of a high T2 lesion load,^11–13^ infratentorial and spinal cord lesions,^14–17^ and the presence of gadolinium-enhancing lesions^16,18^ associate with faster accrual of disability. These findings are supported by recent pathological data, showing a swift accumulation of MS disability associated with a higher lesion load, particularly in the brainstem and spinal cord.^19^

Suppressing relapses and accumulation of MRI biomarkers benefits people with MS in terms of disability accumulation as measured with the Expanded Disability Status Scale (EDSS) score. A reduced accumulation of disability at the group level has been shown as primary and secondary endpoints in pivotal clinical trials for multiple registered MS DMTs versus active comparators or placebo in relapsing MS, including sphingosine-1-phosphate receptor modulators (fingolimod, 6-month confirmed 11.5%–12.5% vs 19.0%^20^ and next-generation compounds), natalizumab (3-month confirmed 17% vs 29%^21^), anti-CD20 monoclonals (ocrelizumab, 24-week confirmed 6.9% vs 10.5%,^22^ ofatumumab, 6-month confirmed 8.1% vs 12.0%^23^), and immune reconstitution therapies (cladribine, 3-month confirmed 14.3%–15.1% vs 20.6%^24^ and alemtuzumab, 6-month confirmed 13% vs 20%^25^) and has been suggested in highly effective DMT-resistant MS cases treated with autologous hematopoietic stem cell transplantation^26^. In the natural history of MS, relapse activity and incidence of associated MRI biomarkers decline with increasing age.^27,28^ A meta-analysis on the effectiveness of classical DMTs to suppress relapses and prevent disability accumulation estimated the most pronounced benefits for younger individuals.^29^ Along this line, a review of clinical trial data detected uncertain benefits in the prevention of disability accumulation in older randomized controlled trial participants.^30^ In an aging real-world cohort, a reduced benefit of high-efficacy therapies has been shown.^31^ Therefore, early prevention of acute focal inflammation appears to be key to prevent disability accumulation in MS. In this context, a long-standing debate of the MS field is whether an escalation approach (starting with first-line therapies and escalating with breakthrough disease) or an induction approach (starting with highly effective therapies and de-escalating when there are safety concerns or long-term stable disease) would provide most benefits for people with MS in the long run.^32^ We modeled for the Dutch situation that earlier application of highly effective registered DMTs will result in a higher health gain with likely similar cost-effectiveness compared to escalation sequences starting with platform therapies.^33^ In line, most published data from prospective studies are in favor of early treatment with highly effective therapies, based on superior reduction in disability accrual in the induction arms.^32^ Randomized clinical trials generating controlled data addressing this issue are still ongoing. We argue that upcoming novel MS therapies make the case for early treatment of relapses and MRI activity with highly effective DMTs even stronger.

## The relevance of suppressing disability progression without acute focal inflammatory activity with disease-modifying therapies

The efficacy of current DMTs in suppressing progressive disability worsening independent of MS relapses or new MRI lesion formation is less certain. Progressive worsening of walking ability, as reflected by higher ranges of the EDSS score without clinically evident relapses, is mostly seen in people with MS from the age of 45 years,^6^ regardless of prior relapses. This is referred to as progressive MS.^2^ Progressive MS without preceding relapses is referred to as primary progressive MS, whereas progressive MS with a history of relapses is referred to as secondary progressive MS.^34^ People with progressive MS reach disability milestones much faster after first presentation.^6,35–37^ Especially in the younger age spectrum, some patients display relapses and new MRI lesion accumulation while also showing progressive disability.^38^ Suppression of this acute focal inflammatory activity associates with the benefit of classical DMTs in progressive MS. In the ORATORIO trial in primary progressive MS, exploratory sub-group analysis revealed that the effectivity of ocrelizumab in preventing 12-week confirmed disability progression was most prominent in participants with at least one gadolinium-enhancing lesion at baseline,^39^ whereas this endpoint was numerically most prominently reached in the EXPAND trial by siponimod-treated participants with secondary progressive MS and recorded relapses within 2 years prior to enrolment.^40^ It should be noted that, in these positive trials, the 12-week confirmed disability worsening endpoint was met by not more than 26%–32.9% of participants in the intervention groups.

Although disability progression without relapses is a dominant feature in longstanding MS, signs of disability progression can be detected early in the course of MS upon careful examination of neurological functioning. In interferon beta-treated participants with relapsing MS of the OPERA trials, 24-week confirmed disability worsening events occurred during 96-week follow-up in 4.8% in temporal relationship with relapses (relapse-associated worsening (RAW)), while 18.2% of these events were independent of relapses (progression independent of relapse activity (PIRA)).^41^ For ocrelizumab-treated participants in these trials, this was 2.1% versus 14.4%, respectively. In the prospective Barcelona cohort following people after a first demyelinating event, disability worsening independent of relapses was noted in 66% of participants during a median follow-up time of 10.5 years, whereas only in 34% of participants worsening was exclusively associated with the occurrence of relapses.^42^ In 8% of these study participants, worsening neurological disability without relapses occurred within the first 5 years after the initial attack. Different definitions for RAW and PIRA have been proposed, which affect the distribution of these events in different cohorts.^43^ However, regardless of definition, it remains uncertain if underlying subclinical acute focal inflammatory activity contributes to PIRA and thus if PIRA is an entirely distinct biological phenomenon. MRI activity is far more prevalent than clinical relapses and is not taken into account in the PIRA definition, and lower rates of PIRA have been found in study arms treated with drugs more effective in suppressing gadolinium-enhancing lesions and new T2 lesions on MRI.^41^ A more recent definition of progression independent of relapses and MRI activity has been proposed to take this limitation into account.^44^ However, this definition remains incomplete to a certain extent as most monitoring protocols focus on brain MRI lesions without taking the spinal cord into account. Some evidence suggests that additional monitoring of the spinal cord may reveal brain-independent silent lesion formation, which could be significant for disability accumulation.^17^

## The opportunities for suppressing disability progression without pronounced suppression of acute focal inflammatory activity with novel therapies

The massive interest of pharmaceutical companies in BTK inhibition resulted in several landmark studies in relapsing and progressive MS.^3^ In the evolutionRMS 1 and 2 trials in people with active relapsing MS, treatment with evobrutinib was compared to treatment with teriflunomide. The primary endpoint, annualized relapse rate, was investigated during the 156-week follow-up, with confirmed disability progression as a secondary endpoint.^45^ Participants all displayed symptoms or MRI signs of focal inflammatory activity within 1 year prior to inclusion, and a secondary progressive MS diagnosis was present in 3.9%. In the evolutionRMS studies, no benefit on relapses could be shown for BTK inhibitor evobrutinib compared to teriflunomide, with a numerically higher number of gadolinium-enhancing MRI lesions in the evobrutinib compared to the teriflunomide group.^45^ The secondary 24-week confirmed disability progression endpoint was similarly distributed, with 6.0% of participants in the evobrutinib group compared to 5.8% of participants in the teriflunomide group reaching an event until week 156. In the Gemini 1 and 2 studies, the efficacy of tolebrutinib against teriflunomide in suppressing relapses and MRI activity was investigated in a cohort of relapsing MS participants with active disease.^4^ The primary endpoint was annualized relapse rate with confirmed disability progression as a secondary endpoint. Participants all displayed symptoms of acute focal inflammation within 2 years or a gadolinium-enhancing lesion within 1 year prior to inclusion; a secondary progressive MS diagnosis was reported in 0.9%. Similar to evobrutinib, there was no benefit on the primary endpoint, annualized relapse rate, compared to teriflunomide, and the number of gadolinium-enhancing lesions was numerically higher in the tolebrutinib compared to the teriflunomide arm. Nevertheless, in the pooled analysis, 8.3% of participants had a 6-month confirmed disability worsening in the tolebrutinib groups, compared to 11.3% in the teriflunomide group, resulting in a 29% reduction in 6-month clinically confirmed disability progression in the tolebrutinib arm. In the Hercules trial, tolebrutinib was compared to placebo in efficacy to suppress disability worsening in non-relapsing participants with secondary progressive MS.^5^ The primary endpoint was confirmed disability progression, with secondary MRI endpoints. Importantly, participants in Hercules were selected to display documented signs of disability progression within 1 year prior to baseline. None of the participants experienced relapses prior to screening, and 12.7% showed at least one gadolinium-enhancing MRI lesion at baseline. This is numerically lower than the 35.3%–37.9% of participants with an active baseline MRI scan, as included in the evolutionRMS^45^ and 32.4%–38.4% of participants with gadolinium-enhancing lesions on the baseline scan, as included in the Gemini trials.^4^ In line with Gemini, this study reported 6-month confirmed disability worsening in 22.6% of participants in the tolebrutinib group compared to 30.7% in the placebo group, resulting in a 31% reduction in 6-month clinically confirmed disability progression in the tolebrutinib group.^5^ This coincided with a statistically significant but modest reduction in the annualized rate of new or expanding T2 lesions on MRI, of which the relative contribution of suppression of new T2 lesion formation as an acute focal inflammation-associated MRI finding versus slowly expanding lesion (SEL) development as a progression-associated finding is not reported.^46^ Annualized relapse was comparably low in both groups (0.033 in the tolebrutinib and 0.032 in the placebo group).

Although the tested BTK inhibitors are, at best, equally efficient as teriflunomide in suppressing relapses in relapsing/MRI-active MS study participants, these observations consistently show that tolebrutinib does delay confirmed progression of disability compared to teriflunomide in relapsing/MRI-active MS and compared to placebo in non-relapsing MS. Since teriflunomide was superior to placebo in preventing relapses in its phase III trial in relapsing MS,^47^ a similar effect of BTK inhibition on these endpoints can be postulated. However, the numerical inferiority of BTK inhibition in preventing gadolinium-enhancing MRI lesions in participants with relapsing MS^4,45^ suggests a putative disadvantage of BTK inhibition compared to teriflunomide in suppressing the acute focal inflammatory component of MS. Of note, teriflunomide was less effective in suppressing relapses in phase III trials as an active comparator to anti-CD20 monoclonal antibodies, ofatumumab and ublituximab, ^23,48^ and sphingosine-1-phosphate receptor modulator ponesimod in relapsing MS.^49^ The difference in effects on confirmed disability progression between evobrutinib and tolebrutinib is not fully understood. Reported proportions of confirmed disability progression were numerically lower in the teriflunomide arm of evolutionRMS compared to Gemini (5.8% of participants with an event versus 11.3% of participants with a multiple imputation-based event, respectively). Since BTK inhibitors modulate B- and T-cell interaction and microglial activation, as takes place locally in the central nervous system in the context of MS pathology,^50,51^ the increased cerebrospinal fluid penetrance of tolebrutinib compared to evobrutinib, as has been reported in preclinical studies, may also contribute to this difference.^52^ The disconnection between effects of tolebrutinib on the occurrence of acute focal inflammation on the one hand and accumulation of disability on the other hand provides convincing evidence that it is possible to target the latter biological component of MS with brain-penetrant compounds. Several other clinical trials are ongoing, targeting specifically non-relapsing progressive MS.

## Allocation of people with MS to novel treatments

**Figure 1.**
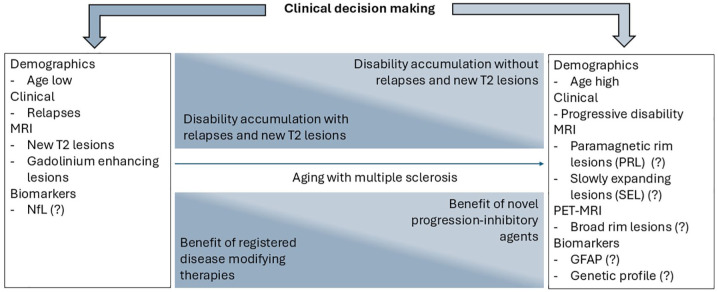
Distribution of clinical events, putatively associated discriminators, and hypothetical benefit of treatment strategies across the lifespan with MS. Biomarkers with an uncertain yet to be consolidated value are indicated with a question mark (?).

The enlarging therapeutic window for non-relapsing progressive MS raises an interesting yet challenging issue: who are the patients who could benefit from these drugs without risking suboptimal control of clinical and radiological activity? Clinical characteristics can be helpful. Clearly, a prior history of disability accumulation without relapses could be a useful indicator. Prognostic scores predict a higher odds of remaining relapse-free after cessation of interferon beta and glatiramer acetate in individuals with relapsing MS free of new lesions on MRI and relapses in the previous 8 years.^53^ Likewise, the recent DISCOMS and DOT-MS studies suggest that the majority of people with long-term stable MS on platform DMTs remain free of short-term relapses and MRI activity after cessation of these therapies.^54,55^ A major component in these prognostic scores is age, as it is associated with a reduced occurrence of relapses and MRI activity and a reduced effectiveness of MS therapies as discussed above. Therefore, a higher age is likely to characterize people with MS with an *a priori* lower odds of benefiting from classical DMTs. Although the risk of undertreating MS activity with BTK inhibition is probably low in this specific age group, there is insufficient data to assess the benefit from novel treatments such as BTK inhibition across the lifespan. Nevertheless, older age groups will most likely be enriched for the non-relapsing progressive MS populations, as they were recruited in the clinical trials (Figure 1). Traditional DMT escalation strategies moving from low, medium to highly effective drugs when it comes to suppressing focal inflammation might therefore be unnecessarily time-consuming in individuals prone to progression. To extend the window of detecting individuals benefiting from therapies such as BTK inhibition, early and aggressive treatment of relapses, and novel MRI lesions will become even more essential. Fine-grained clinimetric tests may assist in identifying patients with progressive neurological disability from early in their disease onwards. However, clinical endpoints will always remain a composite endpoint of many different underlying processes. Hence, they are likely not sufficiently specific to differentiate between disability due to ongoing acute focal inflammation and due to compartmentalized pathology, especially not in a short to medium time span.

Imaging and soluble biomarkers would be helpful to identify eligible people with MS for tolebrutinib or similar other novel therapies. The absence of correlates of ongoing, significant acute focal inflammatory activity will be critical, like new or enhancing MRI lesion formation. On the other hand, positive imaging signs of compartmentalized inflammatory processes may also help in selecting appropriate candidates, as is suggested by several preliminary findings. Presence of four or more paramagnetic rim lesions on susceptibility-weighted images on MRI associated with progressive disability and a 54% reduced risk of 6-month confirmed disability worsening in the tolebrutinib compared to placebo arms of the Hercules trial, and 49% risk reduction in the tolebrutinib compared to teriflunomide arms of the Gemini trials, whereas no differences between arms could be shown for participants lacking paramagnetic rim lesions.^56^ In observational studies, SEL and paramagnetic rim lesions are MRI events associated with disability progression.^57,58^ Likewise, translocator protein (TSPO) active rim lesions on PET-MRI predict future disability accumulation in MS.^59^ Especially, lesions with broad TSPO active rims have been identified as a biomarker of a severe MS disease course.^19^ For blood biomarkers, glial fibrillary acidic protein (GFAP) levels are associated with future disability progression in MS, with high neurofilament light chain (NfL) correlating with acute focal inflammatory activity.^60–62^ The prognostic value of selecting participants based on high serum GFAP and low NfL should become clear from studying clinical trial cohorts. Novel biomarkers can be identified by fundamental and translational research, dissecting modifiable mechanisms driving MS independently of relapses and new lesion formation.^2^ About 80% of people with MS display lesions with active human leukocyte antigen (HLA-)positive rims of myeloid cells upon autopsy,^63^ with broad rims being identified as a histological biomarker for a severe MS course during life.^19^ Dissecting underlying processes may reveal novel molecular pathways translating to clinically useful biomarkers. Although only the tip of the iceberg has recently been identified, genetic variants associated with MS severity and progression may identify people who would benefit most from targeting specific pathophysiological components of disability progression. Although low in minor allele frequency, carriership of the recently identified rs10191329^AA^ severity locus is associated with a younger age of walking with a unilateral aid,^64^ steeper increase in disability accumulation with higher serum NfL levels,^65^ an increased rate of brain atrophy^66^ and a higher rate of gray and white matter lesions coinciding with increased inflammation and axonal loss.^67^ A more complete view of the genetic architecture of MS severity and progression could result in composite biomarkers to putatively aid in prognostication and treatment allocation in MS.

## Practical treatment approaches in MS

The upcoming novel MS diagnostic criteria will likely enable an even earlier diagnosis of MS.^68^ An early effective control of acute focal inflammatory activity as reflected by relapses and new/enhancing lesions on MRI may prove, next to its already known superior effect on prevention of disability accumulation, also as most attractive strategy to extend the window for early use of BTK inhibition. This notion reinforces the existing calls for early treatment of people with MS with highly effective therapies and granular longitudinal assessment of neurological functioning. Patients experiencing quantifiable progressive disability despite reaching a state free of relapses and inflammatory MRI lesions could be eligible candidates for BTK inhibition. Practically, attractive approaches could be to treat early with immune reconstitution therapies (cladribine and alemtuzumab), or in highly active, DMT-resistant cases, autologous hematopoietic stem cell transplantation, followed by novel therapies as BTK-inhibition after immune reconstitution. After a limited number of cycles with anti-CD20 antibodies, currently available data also suggest a subsequent prolonged disease activity-free interval, which could provide a window for early use of BTK inhibition.^69^ This prolonged effect on disease activity might be driven by the long-term reduced memory B compartment despite B-cell repopulation.^26,70^ However, this could also be the weakness of this immune reconstitution therapy-BTK inhibitor sequencing strategy, as these individuals have not been specifically included in Gemini and Hercules trials, and it could theoretically result in a higher infection rate when starting BTK inhibition. Therefore, prospective studies will be needed to evaluate the safety and efficacy of these approaches. In older individuals with limited acute focal inflammatory activity, one could consider starting upfront with BTK inhibition, as this age group has been defined by lower levels of acute focal inflammatory activity, yet the specific efficacy of BTK inhibition in this group has not been scrutinized. Highly effective therapies with a high risk of rebound, such as natalizumab or fingolimod, are less favorable options for a direct transfer toward compounds without profound anti-inflammatory effects. In these cohorts, a switch toward novel compounds via treatment with anti-CD20 could offer a useful strategy.^71,72^ Lastly, first-line platform therapies would, with favorable prognostic factors predicting a low chance of renewed disease activity after cessation,^53^ also allow a transfer toward progression-inhibiting therapies. Especially since the control of relapses by, for instance, BTK inhibition was similar to teriflunomide in phase III trials.^4,45^ However, since this status is usually reached after a prolonged disease course of MS at an older age, the window to benefit from novel treatments will be significantly delayed and shortened. Along these lines, future clinical studies may explore the benefits of combination therapies suppressing both inflammatory and progressive components of MS. Yet, this is currently uncharted territory.

## Concluding remarks

Upcoming novel MS therapies, such as BTK inhibition, targeting MS progression largely independent of relapses, and new MRI lesion formation, offer an opportunity to target a part of MS pathophysiology that has been out of reach until now. We emphasize the need to offer adequate and early treatment of relapses and MRI activity to people with MS, and argue that an aggressive and pro-active treatment of this part of MS may, in the currently changing MS treatment landscape, also drastically expand and advance the window for patients to benefit from these novel therapies. Identification and validation of better imaging and soluble biomarkers tagging specifically both mechanisms may aid in clinical decision-making.

## Declarations

**Ethics approval and consent to participate:** No original data were generated, and no original individual-level data were reused for this work.

**Consent for publication:** No original data were generated, and no original individual-level data were reused for this work.

**Availability of data and materials:** No original data were generated, and no original individual-level data were reused for this work.

## References

[1] Tintore M Vidal-Jordana A Sastre-Garriga J. Treatment of multiple sclerosis - success from bench to bedside. Nat Rev Neurol 2019; 15(1): 53–58. 30315270 10.1038/s41582-018-0082-z

[2] Kuhlmann T Moccia M Coetzee T , et al. Multiple sclerosis progression: time for a new mechanism-driven framework. Lancet Neurol 2023; 22(1): 78–88. 36410373 10.1016/S1474-4422(22)00289-7 PMC10463558

[3] Airas L Bermel RA Chitnis T , et al. A review of Bruton’s tyrosine kinase inhibitors in multiple sclerosis. Ther Adv Neurol Disord 2024; 17: 17562864241233041. 10.1177/17562864241233041 PMC11025433 38638671

[4] Oh JW Arnold DL Cree Bac , et al. Tolebrutinib versus teriflunomide in relapsing multiple sclerosis. N Engl J Med 2025; 392(19): 1893–1904. 40202623 10.1056/NEJMoa2415985

[5] Fox RJ Bar-Or A Traboulsee A , et al. Tolebrutinib in nonrelapsing secondary progressive multiple sclerosis. N Engl J Med 2025; 392(19): 1883–1892. 40202696 10.1056/NEJMoa2415988

[6] Leray E Yaouanq J Le Page E , et al. Evidence for a two-stage disability progression in multiple sclerosis. Brain 2010; 133(Pt 7): 1900–1913. 20423930 10.1093/brain/awq076 PMC2892936

[7] Scalfari A Neuhaus A Degenhardt A , et al. The natural history of multiple sclerosis, a geographically based study 10: relapses and long-term disability. Brain 2010; 133(Pt 7): 1914–1929. 20534650 10.1093/brain/awq118 PMC2892939

[8] Vasconcelos CCF Aurenção JCK Thuler LCS , et al. Prognostic factors associated with long-term disability and secondary progression in patients with multiple sclerosis. Mult Scler Relat Disord 2016; 8: 27–34. 27456871 10.1016/j.msard.2016.03.011

[9] Sormani MP Gasperini C Romeo M , et al. Assessing response to interferon-β in a multicenter dataset of patients with MS. Neurology 2016; 87(2): 134–140. 27306626 10.1212/WNL.0000000000002830

[10] Amato MP Ponziani G Bartolozzi ML , et al. A prospective study on the natural history of multiple sclerosis: clues to the conduct and interpretation of clinical trials. J Neurol Sci 1999; 168(2): 96–106. 10526190 10.1016/s0022-510x(99)00143-4

[11] Fisniku LK Brex PA Altmann DR , et al. Disability and T2 MRI lesions: a 20-year follow-up of patients with relapse onset of multiple sclerosis. Brain 2008; 131(Pt 3): 808–817. 18234696 10.1093/brain/awm329

[12] Swanton JK Fernando KT Dalton CM , et al. Early MRI in optic neuritis: the risk for disability. Neurology 2009; 72: 542–550. 19204264 10.1212/01.wnl.0000341935.41852.82

[13] Tintore M Rovira À Río J , et al. Defining high, medium and low impact prognostic factors for developing multiple sclerosis. Brain 2015; 138(Pt 7): 1863–1874. 25902415 10.1093/brain/awv105

[14] Minneboo A Barkhof F Polman CH , et al. Infratentorial lesions predict long-term disability in patients with initial findings suggestive of multiple sclerosis. Arch Neurol 2004; 61(2): 217–221. 14967769 10.1001/archneur.61.2.217

[15] Tintore M Rovira A Arrambide G , et al. Brainstem lesions in clinically isolated syndromes. Neurology 2010; 75(21): 1933–1938. 21098409 10.1212/WNL.0b013e3181feb26f

[16] Brownlee WJ Altmann DR Prados F , et al. Early imaging predictors of long-term outcomes in relapse-onset multiple sclerosis. Brain 2019; 142(8): 2276–2287. 31342055 10.1093/brain/awz156

[17] Keegan BM Absinta M Cohen-Adad J , et al. Spinal cord evaluation in multiple sclerosis: clinical and radiological associations, present and future. Brain Commun 2024; 6(6): fcae395. 10.1093/braincomms/fcae395 PMC11604059 39611182

[18] Sormani MP Bruzzi P. MRI lesions as a surrogate for relapses in multiple sclerosis: a meta-analysis of randomised trials. Lancet Neurol 2013; 12(7): 669–676. 23743084 10.1016/S1474-4422(13)70103-0

[19] Klotz L Smolders J Lehto J , et al. Broad rim lesions are a novel pathological and imaging biomarker for rapid disease progression in Multiple Sclerosis. Nat Med 2025; 31(6): 2016–2026. 40301560 10.1038/s41591-025-03625-7 PMC12176629

[20] Kappos L Radue EW O’Connor P . A placebo-controlled trial of oral fingolimod in relapsing multiple sclerosis. N Engl J Med 2010; 362(5): 387–401. 20089952 10.1056/NEJMoa0909494

[21] Polman C O’Connor PW Havrdova E , et al. A randomized, placebo-controlled trial of natalizumab for relapsing multiple sclerosis. N Engl J Med 2006; 354(9): 899–910. 16510744 10.1056/NEJMoa044397

[22] Hauser SL Bar-Or A Comi G , et al. Ocrelizumab versus Interferon beta-1a in relapsing multiple sclerosis. N Engl J Med 2017; 376(3): 221–234. 28002679 10.1056/NEJMoa1601277

[23] Hauser SL Bar-Or A Cohen JA , et al. Ofatumumab versus teriflunomide in multiple sclerosis. N Engl J Med 2020; 383(6): 546–557. 32757523 10.1056/NEJMoa1917246

[24] Giovannoni G Comi G Cook S , et al. A placebo-controlled trial of oral cladribine for relapsing multiple sclerosis. N Engl J Med 2010; 362(5): 416–426. 20089960 10.1056/NEJMoa0902533

[25] Coles A Twyman CL Arnold DL , et al. Alemtuzumab for patients with relapsing multiple sclerosis after disease-modifying therapy: a randomised controlled phase 3 trial. Lancet 2012; 380(9856): 1829–1839. 23122650 10.1016/S0140-6736(12)61768-1

[26] Muraro PA Mariottini A Greco R , et al. Autologous haematopoietic stem cell transplantation for treatment of multiple sclerosis and neuromyelitis optica spectrum disorder - recommendations from ECTRIMS and the EBMT. Nat Rev Neurol 2025; 21(3): 140–158. 39814869 10.1038/s41582-024-01050-x

[27] Coerver E Janssens S Ahmed A , et al. Association between age and inflammatory disease activity on magnetic resonance imaging in relapse onset multiple sclerosis during long-term follow-up. Eur J Neurol 2023; 30(8): 2385–2392. 37170817 10.1111/ene.15862

[28] Tremlett H Zhao Y Rieckmann P , et al. New perspectives in the natural history of multiple sclerosis. Neurology 2010; 74(24): 2004–2015. 20548045 10.1212/WNL.0b013e3181e3973f

[29] Weideman AM Tapia-Maltos MA Johnson K , et al. Meta-analysis of the age-dependent efficacy of multiple sclerosis treatments. Front Neurol 2017; 10: 00577. 10.3389/fneur.2017.00577 PMC5686062 29176956

[30] Jakimovski D Eckert SP Zivadinov R , et al. Considering patient age when treating multiple sclerosis across the adult lifespan. Expert Rev Neurother 2021; 21(3): 353–364. 33595379 10.1080/14737175.2021.1886082

[31] Vollmer BL Wolf AB Sillau S , et al. Evolution of disease modifying therapy benefits and risks: an argument for de-escalation as a treatment paradigm for patients with multiple sclerosis. Front Neurol 2022; 12: 799138. 35145470 10.3389/fneur.2021.799138 PMC8821102

[32] Bou Rjeily N Mowry E . Treating early relapsing multiple sclerosis: induction & escalation approaches. Pract Neurol 2022; 22(1): 39–43.

[33] Smets I Versteegh M Huygens S , et al. Benefits of early highly effective versus escalation treatment strategies in relapsing multiple sclerosis estimated using a treatment-sequence model. Mult Scler 2024; 30(8): 1016–1025. 38859625 10.1177/13524585241258692 PMC11290018

[34] Lublin FD Reingold SC Cohen JA , et al. Defining the clinical course of multiple sclerosis: the 2013 revisions. Neurology 2014; 83(3): 278–286. 24871874 10.1212/WNL.0000000000000560 PMC4117366

[35] Confavreux C. Early clinical predictors and progression of irreversible disability in multiple sclerosis: an amnesic process. Brain 2003; 126(Pt4): 770–782. 12615637 10.1093/brain/awg081

[36] Menon S Shirani A Zhao Y , et al. Characterising aggressive multiple sclerosis. J Neurol Neurosurg Psychiatry 2013; 84(11): 1192–1198. 23744892 10.1136/jnnp-2013-304951

[37] Traboulsee AL Cornelisse P Sandberg-Wollheim M , et al. Prognostic factors for long-term outcomes in relapsing–remitting multiple sclerosis. Mult Scler J Exp Transl Clin 2016; 2: 2055217316666406. 10.1177/2055217316666406 PMC5433509 28607737

[38] Blok KM van Rosmalen J Tebayna N , et al. Disease activity in primary progressive multiple sclerosis: a systematic review and meta-analysis. Front Neurol 2023; 14: 1277477. 38020591 10.3389/fneur.2023.1277477 PMC10661414

[39] Montalban X Hauser SL Kappos L , et al. Ocrelizumab versus placebo in primary progressive multiple sclerosis. N Engl J Med 2017; 376(3): 209–220. 28002688 10.1056/NEJMoa1606468

[40] Kappos L Bar-Or A Cree BAC , et al. Siponimod versus placebo in secondary progressive multiple sclerosis (EXPAND): a double-blind, randomised, phase 3 study. Lancet 2018; 391(10127): P1263–P1273. 10.1016/S0140-6736(18)30475-6 29576505

[41] Kappos L Wolinsky JS Giovannoni G , et al. Contribution of relapse-independent progression vs relapse-associated worsening to overall confirmed disability accumulation in typical relapsing multiple sclerosis in a pooled analysis of 2 randomized clinical trials. JAMA Neurol 2020; 77(9): 1132–1140. 32511687 10.1001/jamaneurol.2020.1568 PMC7281382

[42] Tür C Carbonell-Mirabent P Cobo-Calvo A , et al. Association of early progression independent of relapse activity with long-term disability after a first demyelinating event in multiple sclerosis. JAMA Neurol 2023; 80(2): 151–160. 36534392 10.1001/jamaneurol.2022.4655 PMC9856884

[43] Müller J Sharmin S Lorscheider J , et al. Standardized definition of progression independent of relapse activity (PIRA) in relapsing-remitting multiple sclerosis. JAMA Neurol 2025; 82(6): 614–625. 40227706 10.1001/jamaneurol.2025.0495 PMC11997854

[44] Ciccarelli O Brakhof F Calabrese M , et al. Using the progression independent of relapse activity framework to unveil the pathobiological foundations of multiple sclerosis. Neurology 2024; 103(1): e209444. 10.1212/WNL.0000000000209444 PMC11226318 38889384

[45] Montalban X Vermersch P Arnold DL , et al. Safety and efficacy of evobrutinib in relapsing multiple sclerosis (evolutionRMS1 and evolutionRMS2): two multicentre, randomised, double-blind, active-controlled, phase 3 trials. Lancet Neurol 2024; 23(11): 1119–1132. 39307151 10.1016/S1474-4422(24)00328-4

[46] Elliott C Wolinsky JS Hauser SL , et al. Slowly expanding/evolving lesions as a magnetic resonance imaging marker of chronic active multiple sclerosis lesions. Mult Scler 2019; 25(14): 1915–1925. 30566027 10.1177/1352458518814117 PMC6876256

[47] Confavreux C O’Connor P Comi G , et al. Oral teriflunomide for patients with relapsing multiple sclerosis (TOWER): a randomised, double-blind, placebo-controlled, phase 3 trial. Lancet Neurol 2014; 13(3): 247–256. 24461574 10.1016/S1474-4422(13)70308-9

[48] Steinman L Fox E Hartung HP , et al. Ublituximab versus teriflunomide in relapsing multiple sclerosis. N Engl J Med 2022; 387(8): 704–714. 36001711 10.1056/NEJMoa2201904

[49] Kappos L Fox RJ Burcklen M , et al. Ponesimod compared with teriflunomide in patients with relapsing multiple sclerosis in the active-comparator phase 3 OPTIMUM study: a randomized clinical trial. JAMA Neurol 2021; 78(5): 558–567. 33779698 10.1001/jamaneurol.2021.0405 PMC8008435

[50] Rijvers L van Langelaar J Bogers L , et al. Human T-bet+ B cell development is associated with BTK activity and suppressed by evobrutinib. JCI Insight 2022; 7(16): e160909. 10.1172/jci.insight.160909 PMC9462504 35852869

[51] Gruber RC Wirak GS Blazier AS , et al. BTK regulates microglial function and neuroinflammation in human stem cell models and mouse models of multiple sclerosis. Nature Commun 2024; 15: 10116. 39578444 10.1038/s41467-024-54430-8 PMC11584639

[52] Turner TJ Brun P Gruber RC , et al. Comparative CNS pharmacology of the Bruton’s tyrosine kinase (BTK) inhibitor tolebrutinib versus other BTK inhibitor candidates for treating multiple sclerosis. Drugs R D 2024; 24(2): 263–274. 38965189 10.1007/s40268-024-00468-4 PMC11315827

[53] Bsteh G Hegen H Riedl K , et al. Quantifying the risk of disease reactivation after interferon and glatiramer acetate discontinuation in multiple sclerosis: the VIAADISC score. Eur J Neurol 2021; 28(5): 1609–1616. 33370478 10.1111/ene.14705 PMC8248019

[54] Coerver EME Fung WH de Beukelaar J , et al. Discontinuation of first-line disease-modifying therapy in patients with stable multiple sclerosis: the DOT-MS randomized clinical trial. JAMA Neurol 2025; 82(2): 123–131. 39652340 10.1001/jamaneurol.2024.4164 PMC11811793

[55] Corboy JR Fox RJ Kister I , et al. Risk of new disease activity in patients with multiple sclerosis who continue or discontinue disease-modifying therapies (DISCOMS): a multicentre, randomised, single-blind, phase 4, non-inferiority trial. Lancet Neurol 2023; 22(7): 568–577. 37353277 10.1016/S1474-4422(23)00154-0

[56] Oh J Fox RJ Arnold DL , et al. LB1.1. Paramagnetic rim lesions as a prognostic and predictive biomarker in the tolebrutinib phase 3 trials for disability outcomes. Presented at ACTRIMS 2025, February 27–March 1, 2025, West Palm Beach, Florida.

[57] Borrelli S Martire MS Stölting A , et al. Central vein sign, cortical lesions, and paramagnetic rim lesions for the diagnostic and prognostic workup of multiple sclerosis. Neurol Neuroimmunol Neuroinflamm 2024; 11(4): e200253. 10.1212/NXI.0000000000200253 PMC11129678 38788180

[58] Calvi A Clarke MA Prados F , et al. Relationship between paramagnetic rim lesions and slowly expanding lesions in multiple sclerosis. Mult Scler 2023; 29(3): 352–362. 36515487 10.1177/13524585221141964 PMC9972234

[59] Polvinen E Matilainen M Nylund M , et al. TSPO-detectable chronic active lesions predict disease progression in multiple sclerosis. Neurol Neuroimmunol Neuroinflamm 2023; 10(5): e200133. 10.1212/NXI.0000000000200133 PMC10291892 37349108

[60] Abdelhak A Antweiler K Kowarik MC , et al. Serum glial fibrillary acidic protein and disability progression in progressive multiple sclerosis. Ann Clin Transl Neurol 2023; 11(2): 477–485. 38111972 10.1002/acn3.51969 PMC10863922

[61] Barro C Healy BC Liu Y , et al. Serum GFAP and NfL levels differentiate subsequent progression and disease activity in patients with progressive multiple sclerosis. Neurol Neuroimmunol Neuroinflamm 2022; 10(1): e200052. 10.1212/NXI.0000000000200052 PMC9749933 36376097

[62] Blok KM Klein Kranenbarg RAM Ananth K , et al. Multifaceted biomarkers suggest a similar profile of CNS pathology in relapsing and progressive MS. Eur J Neurol 2025; 32(2): e70052. 10.1111/ene.70052 PMC11795420 39907163

[63] Luchetti S Fransen NL van Eden CG , et al. Progressive multiple sclerosis patients show substantial lesion activity that correlates with clinical disease severity and sex: a retrospective autopsy cohort analysis. Acta Neuropathol 2018; 135(4): 511–528. 29441412 10.1007/s00401-018-1818-y PMC5978927

[64] International Multiple Sclerosis Genetics Consortium, MultipleMS Consortium. Locus for severity implicates CNS resilience in progression of multiple sclerosis. Nature 2023; 619(7969): 323–331. 37380766 10.1038/s41586-023-06250-x PMC10602210

[65] Protopapa M Steffen F Schraad M , et al. Increased disability progression in rs10191329AA carriers with multiple sclerosis is preceded by neurofilament light chain elevations. Ann Neurol 2025; 97(3): 596–605. 39588882 10.1002/ana.27144 PMC11831884

[66] Gasperi C Wiltgen T McGinnis J , et al. A genetic risk variant for multiple sclerosis severity is associated with brain atrophy. Ann Neurol 2023; 94(6): 1080–1085. 37753809 10.1002/ana.26807 PMC11303986

[67] Engelenburg HJ van den Bosch AMR Chen JQA , et al. Multiple sclerosis severity variant in DYSF-ZNF638 locus associates with neuronal loss and inflammation. iScience 2025; 28(5): 112430. 40352730 10.1016/j.isci.2025.112430 PMC12063138

[68] Montalban X. Revised McDonald criteria 2023. Presented at Scientific Session 1, ECTRIMS 2024, 18–20 September 2024, Copenhagen, Denmark.

[69] Smets I Wokke B Smolders J. Should anti-CD20 be used as an immune reconstitution therapy? Mult Scler 2023; 29(2): 308–310. 35822293 10.1177/13524585221109386 PMC9925887

[70] Teschner VE Fleck AK Walter C , et al. Single-cell profiling reveals preferential reduction of memory B cell subsets in cladribine patients that correlates with treatment response. Ther Adv Neurol Disord 2023; 16: 17562864231211077. 10.1177/17562864231211077 PMC10710756 38084102

[71] Zhu C Zhou Z Roos I , et al. Comparing switch to ocrelizumab, cladribine or natalizumab after fingolimod treatment cessation in multiple sclerosis. J Neurol Neurosurg Psychiatry 2022; 93(12): 1330–1337. 36261289 10.1136/jnnp-2022-330104

[72] Zhu C Kalincik T Horakova D , et al. Comparison between dimethyl fumarate, fingolimod, and ocrelizumab after natalizumab cessation. JAMA Neurol 2023; 80(7): 739–748. 37273217 10.1001/jamaneurol.2023.1542 PMC10242509

